# Cerebellar neuronal dysfunction accompanies early motor symptoms in spinocerebellar ataxia type 3

**DOI:** 10.1242/dmm.049514

**Published:** 2022-08-05

**Authors:** Kristin Mayoral-Palarz, Andreia Neves-Carvalho, Sara Duarte-Silva, Daniela Monteiro-Fernandes, Patrícia Maciel, Kamran Khodakhah

**Affiliations:** 1 Dominick P. Purpura Department of Neuroscience, Albert Einstein College of Medicine, Bronx, NY 10461, USA; 2 Life and Health Sciences Research Institute (ICVS), School of Medicine, University of Minho, 4710-057 Braga, Portugal; 3 ICVS/3B's – PT Government Associate Laboratory, 4710-057 Braga/Guimarães, Portugal

**Keywords:** Cerebellum, Ataxia, SCA3, Purkinje cells

## Abstract

Spinocerebellar ataxia type 3 (SCA3) is an adult-onset, progressive ataxia. SCA3 presents with ataxia before any gross neuropathology. A feature of many cerebellar ataxias is aberrant cerebellar output that contributes to motor dysfunction. We examined whether abnormal cerebellar output was present in the CMVMJD135 SCA3 mouse model and, if so, whether it correlated with the disease onset and progression. *In vivo* recordings showed that the activity of deep cerebellar nuclei neurons, the main output of the cerebellum, was altered. The aberrant activity correlated with the onset of ataxia. However, although the severity of ataxia increased with age, the severity of the aberrant cerebellar output was not progressive. The abnormal cerebellar output, however, was accompanied by non-progressive abnormal activity of their upstream synaptic inputs, the Purkinje cells. *In vitro* recordings indicated that alterations in intrinsic Purkinje cell pacemaking and in their synaptic inputs contributed to abnormal Purkinje cell activity. These findings implicate abnormal cerebellar physiology as an early, consistent contributor to pathophysiology in SCA3, and suggest that the aberrant cerebellar output could be an appropriate therapeutic target in SCA3.

## Abstract (precis)

**Summary:** In a mouse model of spinocerebellar ataxia type 3 (SCA3), aberrant cerebellar physiology is apparent early in disease, prior to cerebellar neuronal pathology. Aberrant cerebellar output could be a therapeutic target in SCA3.

## INTRODUCTION

The cerebellum is involved in motor coordination and maintenance of balance. Dysfunction of the cerebellum can lead to ataxia, or uncoordinated movement. The most common dominantly inherited ataxia is spinocerebellar ataxia type 3 (SCA3), also known as Machado–Joseph disease. SCA3 is caused by a heterozygous (CAG)/polyglutamine expansion of the ataxin-3 gene (Kawaguchi et al., 1994). Patients present with adult-onset progressive ataxia, along with a combination of ophthalmoplegia, spasticity, dystonia, muscular atrophy or other extrapyramidal signs (Coutinho and Andrade, 1978; Barbeau et al., 1984). The most common affected regions in pathology include the cerebellar dentate nucleus, pallidum, substantia nigra, thalamus, subthalamic nuclei, red nuclei and, to a lesser extent, cerebellar cortex (Woods and Schaumburg, 1972; Rosenberg et al., 1976; Romanul et al., 1977; Stefanescu et al., 2015; Hernandez-Castillo et al., 2018; Koeppen, 2018; Wang et al., 2020). Degeneration and other pathological hallmarks, such as protein aggregate formation, occur late in the course of the disease.

In the cerebellum of SCA3 patients, pathology has consistently shown late-onset degeneration of the deep cerebellar nuclei (DCN), the main output of the cerebellum, but little to no degeneration of the Purkinje cells, the main computational unit of the cerebellar cortex (Rosenberg et al., 1976; Haines and Dietrichs, 2012; Koeppen, 2018). It is plausible that cerebellar neuronal dysfunction contributes to the early motor phenotype. The cerebellum encodes movement-related information through rapid increases or decreases in the firing rate of the output nuclei, the DCN (Thach, 1968; Eccles, 1973). The DCN neurons are intrinsically active (Raman et al., 2000), meaning that they fire in the absence of synaptic input and fire with a regular firing pattern (Alviña and Khodakhah, 2008). If the precision of spontaneous activity is altered, the accuracy of encoding synaptic information will be impaired. The DCN receive synaptic input from mossy fibers, climbing fibers and Purkinje cells. Purkinje cells are also intrinsically active neurons (Raman and Bean, 1999) and have a regular firing pattern (Häusser and Clark, 1997). Disruption to the regularity of firing of Purkinje cells and the neurons within the DCN has been found in other mouse models of ataxia (Walter et al., 2006; Alviña and Khodakhah, 2010a,b; Tara et al., 2018). As Purkinje cells are the main computational unit of the cerebellar cortex and converge onto the DCN, disruption to their activity can impact the ability of the output of the cerebellum to accurately encode motor-related events. This raises two questions. Is cerebellar neuronal activity altered in SCA3? If so, is the dysfunction progressive, and does it correlate with the motor phenotype?

A SCA3 mouse model (CMVMJD135) expresses the expanded disease-relevant ataxin-3c isoform under the human cytomegalovirus (CMV) promoter in a heterozygous manner, conferring wide expression at near endogenous levels throughout the nervous system, similar to what is seen in patients (Silva-Fernandes et al., 2014; Teixeira-Castro et al., 2015). Overall, the mouse model is ideal to study the cerebellar contribution of disease as it recapitulates the adult-onset, progressive motor phenotypes seen in SCA3 patients, neuropathological findings and late-in-disease neuronal degeneration profile, with sparing of Purkinje cells.

In the CMVMJD135 mouse model (referred to as SCA3 mice throughout this paper), the DCN had irregular firing in awake, head-restrained mice. This indicated early, non-progressive, cerebellar neuronal alteration in SCA3 mice from either DCN neurons, or from irregular activity upstream of the nuclei. *In vivo* Purkinje cell activity, which converges onto the DCN, was also irregular, but not progressive, likely contributing to the abnormal cerebellar output. *In vitro* acute slice recordings allowed investigation of the contributors to Purkinje cell irregular firing. Purkinje cell irregularity had both aberrant intrinsic firing and altered synaptic activity, with a significant synaptic contribution. Altogether, we find that the cerebellum is a site of early dysfunction, before DCN neuronal death or ataxin-3 intranuclear inclusions are detectable, suggesting that Purkinje cell irregular activity may be an early driver of pathophysiology and a potential therapeutic target in SCA3.

## RESULTS

### SCA3 mice exhibit motor dysfunction early in disease

**Fig. 1.**
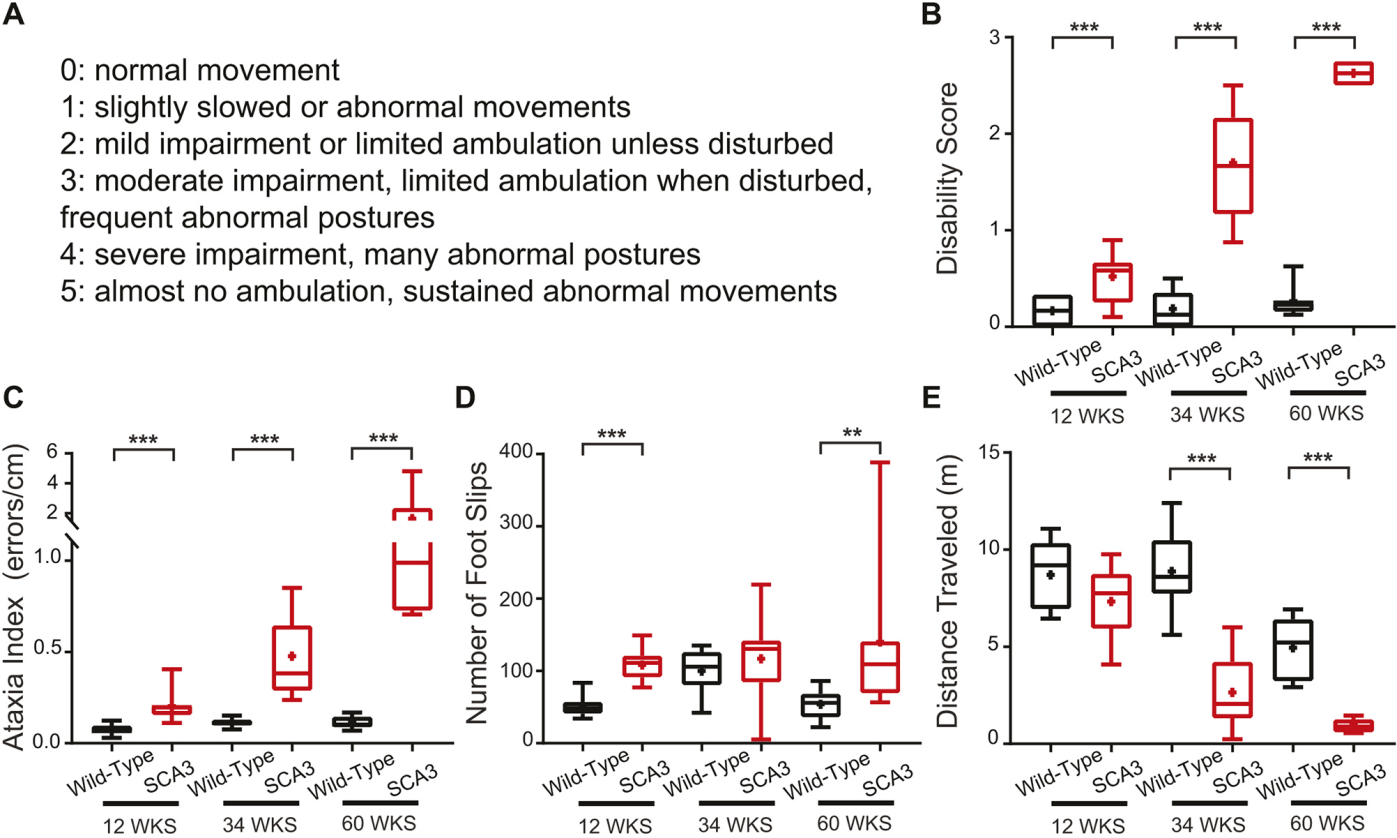
**SCA3 motor symptoms progress with age, with onset as early as 12** **weeks.** (A-E) SCA3 and wild-type mice were tested at 12, 34 and 60 weeks of age on the disability score (A,B) and parallel rod floor test (C-E). SCA3 mice have an increased disability score (A,B) compared to wild types at 12 weeks, and the score progresses with age. SCA3 mice have an increased ataxia index (C) at 12 weeks due to increased foot slips (D), with no change in distance traveled (E). At 34 and 60 weeks of age, the increased ataxia index reflects combined decrease in distance traveled and increased foot slips. Parallel rod floor test: 12 weeks: wild-type *N*=9, SCA3 *N*=9. 34 weeks: wild-type *N*=8, SCA3 *N*=9. 60 weeks: wild-type *N*=8, SCA3 *N*=7. Disability score (all scores are average of two trials): 12 weeks: wild-type *N*=10, SCA3 *N*=15. 34 weeks: wild-type *N*=17, SCA3 *N*=19. 60 weeks: wild-type *N*=8, SCA3 *N*=7. **P<0.01, ***P<0.001. Unpaired, two-tailed Student's *t*-test (B, 12 weeks; D, 12 weeks, 34 weeks; E, all), Mann–Whitney test (B, 34 weeks, 60 weeks; C, all; D, 60 weeks). In all figures, boxes represent the 25-75th percentiles, and the median is indicated. The mean is indicated by a plus sign. The whiskers indicate the 10th and 90th percentiles.

To further characterize the motor phenotype of the SCA3 mice across the course of the disease, mice were assessed using established tests of motor performance not previously applied to this particular disease model at an early, mid and late disease state. The littermate wild types, referred to as wild type throughout this paper, and SCA3 mice were tested on a disability scale for gross motor abnormalities (Weisz et al., 2005; Tara et al., 2018). Using this scale, a mouse with no motor symptoms receives a score of 0, with a score of 1 indicating abnormal gait, and the severity of motor impairment scales up to a score of 5 (Fig. 1A). Based on this assessment, SCA3 mice had motor impairment at 12 weeks of age (wild-type 0.17±0.14, SCA3 0.52±0.28, *P*=0.0018), with overt impairment at 34 weeks (wild-type 0.19±0.17, SCA3 1.70±0.58, *P*<0.0001) and severe impairment by 60 weeks (wild-type 0.27±0.16, SCA3 2.63±0.10, *P*=0.0003) (Fig. 1B). This demonstrates the progressive nature of the disease in SCA3 mice. By 60 weeks, the motor symptoms are a combination of deficits from the central and peripheral nervous systems (Duarte-Silva et al., 2014; Silva-Fernandes et al., 2014; Teixeira-Castro et al., 2015; Esteves et al., 2019).

The second assay, the parallel rod floor test, is more sensitive to motor incoordination and eliminates variability in assessing the disability score due to human error (Kamens et al., 2005; Kamens and Crabbe, 2007). The ataxia index (Fig. 1C), calculated by number of foot slips (Fig. 1D) per distance traveled (cm) (Fig. 1E), was averaged per mouse over two trials. SCA3 mice had a higher ataxia index than wild types as early as 12 weeks (wild-type 0.074±0.029, SCA3 0.202±0.083, *P*<0.0001), with the ataxia index further increasing at 34 weeks (wild-type 0.112±0.023, SCA3 0.476±0.211, *P*<0.0001) and 60 weeks (wild-type 0.111±0.035, SCA3 1.645±1.51, *P*=0.0003) of age (Fig. 1C-E). Interestingly, at 12 weeks, the increased ataxia index was driven by an increased number of foot slips, with no significant decrease in distance traveled, indicating motor incoordination and not an overall change in gross movement as a main contributor to the impairment (Fig. 1D,E). Although the progressive increase in ataxia index was likely due to a combination of increased central and peripheral dysfunction, the parallel rod floor test provides a sensitive measure of motor impairment in SCA3 mice as early as 12 weeks of age.

### DCN do not display neuronal cell death at 22 weeks in SCA3 mice

**Fig. 2.**
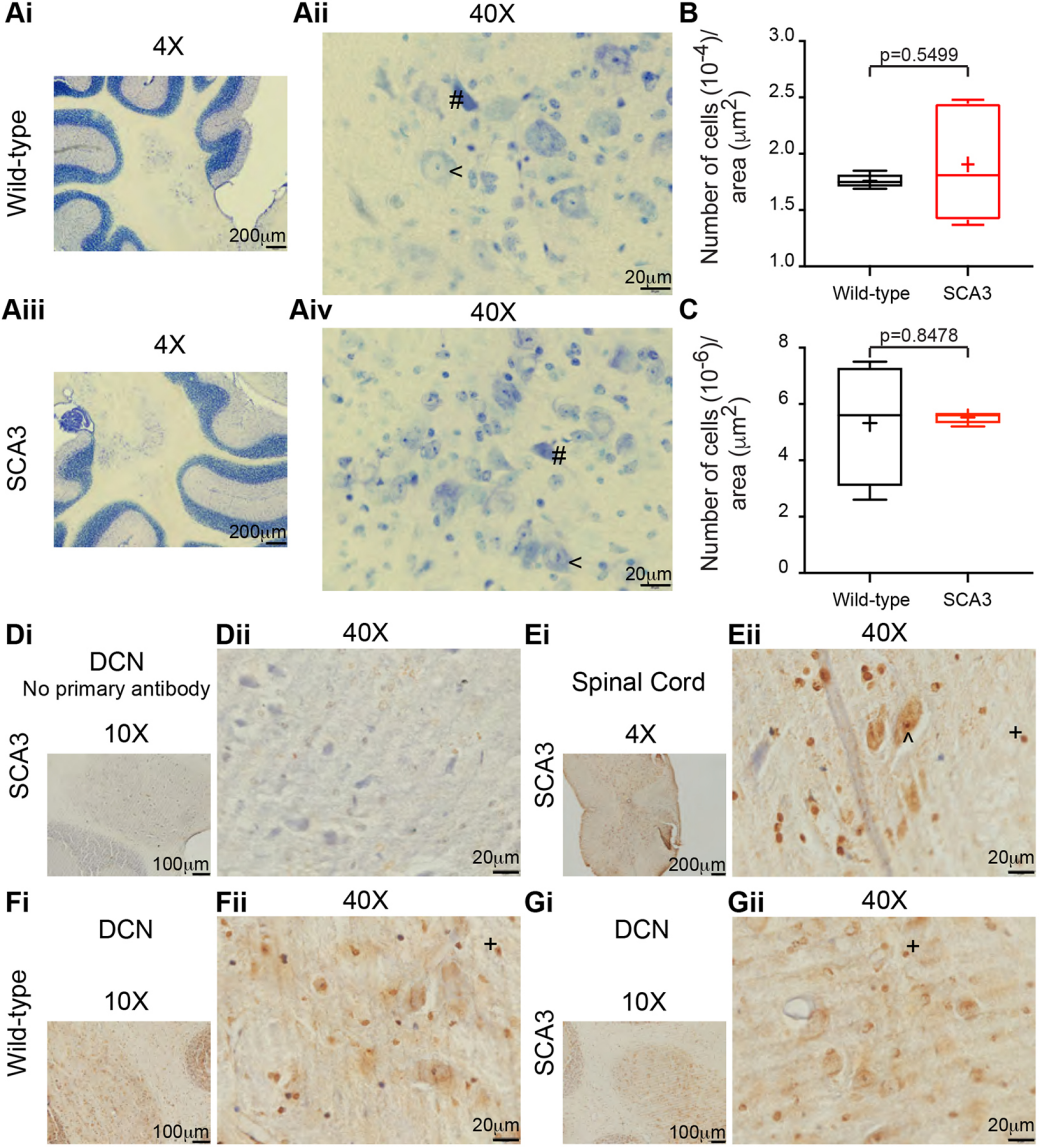
**Lack of deep cerebellar nuclei (DCN) cell death or ataxin-3 intranuclear inclusions at 22** **weeks in SCA3 mice.** (Ai-Gii) The DCN were analyzed for SCA3 hallmark pathology at 22 weeks of age. Cresyl Violet staining of the DCN from wild-type (Ai,Aii) and SCA3 (Aiii,Aiv) mice, with an example of a healthy neuron denoted by a ‘<’ and an example of a pyknotic cell denoted by a ‘#’. There was no difference in the number of heathy neurons (B) or the number of pyknotic cells (C) between wild-type and SCA3 mice. Immunohistochemistry for anti-ataxin-3 (1:500, Millipore, 1H9) was used to detect the presence of intranuclear ataxin-3 inclusions. A negative control, without the primary antibody, demonstrated the background signal from the tissue (Di,Dii). A positive control from the spinal cord of a SCA3 mouse at 22 weeks, with an ataxin-3 intranuclear inclusion denoted by a ‘^’ (Ei,Eii). Example images from the DCN of wild-type (Fi,Fii) and SCA3 (Gi,Gii) mice lacked ataxin-3 intranuclear inclusions. Example glial cells are denoted by a ‘+’. Cytoplasmic ataxin-3 was detected in all images. Cresyl Violet staining: wild-type *N*=4, SCA3 *N*=5; *n*=2-8 sections per animal. Ataxin-3 staining: wild-type DCN *N*=2, *n*=3 sections per animal; SCA3 DCN *N*=5, *n*=3-5 sections per animal. SCA3 spinal cord *N*=1. Negative control, no primary antibody: SCA3 *N*=1 for both DCN and spinal cord. Unpaired, two-tailed Student's *t*-test.

In the SCA3 mice, it has been reported that the DCN neurons have intranuclear ataxin-3 inclusions around 28 weeks, decreased DCN volume around 42 weeks and neuronal cell loss around 56 weeks (Silva-Fernandes et al., 2014; Rodríguez-Cueto et al., 2016; Duarte-Silva et al., 2018; Esteves et al., 2019). Abnormal DCN neuronal pathology or cerebellar neuronal firing changes could contribute to the early cerebellar motor deficits detected here at 12 weeks. To assess DCN neuronal pathology, the number of healthy and pyknotic cells were counted at 22 weeks of age in SCA3 and wild-type mice. The DCN were stained with Cresyl Violet and imaged to visualize the cells (Fig. 2A-C). There was no difference in the number of healthy neurons between wild-type and SCA3 mice (wild-type 1.76×10^−4^±6.25×10^−6^, SCA3 1.91×10^−4^±5.19×10^−5^, *P*=0.5499) (Fig. 2B). There was also no difference in the number of pyknotic cells between wild-type and SCA3 mice (wild-type 5.33×10^−6^±2.22×10^−6^, SCA3 5.52×10^−6^±2.17×10^−7^, *P*=0.8478) (Fig. 2C). Thus, in agreement with what has previously been demonstrated, increased DCN neuronal loss does not occur in SCA3 mice before 22 weeks of age.

One hallmark of SCA3 is the presence of ataxin-3 intranuclear protein inclusions. In the DCN of SCA3 mice, these inclusions have been reported around 28 weeks (Esteves et al., 2019). We assessed 22-week-old SCA3 mice for DCN ataxin-3 intranuclear inclusions. As expected, when no anti-ataxin-3 primary antibody was used, only background signal was detected in the DCN (Fig. 2D). In the spinal cord (used as a positive control), as expected (Silva-Fernandes et al., 2014; Esteves et al., 2019), ataxin-3 intranuclear inclusions were detected in this group of SCA3 mice (Fig. 2E). In the same animals, where ataxin-3 intranuclear inclusions were detected in the spinal cord, there were no inclusions detected in the DCN of SCA3 mice (Fig. 2G). Thus, we demonstrated early motor impairment at 12 weeks (Fig. 1), an age before cerebellar DCN ataxin-3 nuclear aggregates were detected and when there was no increased cell death, pyknosis or other signs of neuropathology (Fig. 2).

### Activity of DCN is altered, but not progressive, in SCA3 mice

We next examined whether there were any alterations in the activity of the cerebellar output nuclei *in vivo*. To minimize the time-variant information encoded by synaptic activity, we assessed the firing of the neurons in the DCN in stationary animals. Although the cerebellar contribution to actions such as standing remained, the need for motor signals from outside the cerebellum that drive rapid changes in firing was reduced, allowing for a more accurate assessment of the precision of firing. Therefore, *in vivo* recordings of the DCN neurons were made from awake, head-restrained, stationary mice. The inter-spike interval (ISI) coefficient of variation (CV) was used to measure the regularity of firing (Häusser and Clark, 1997; Walter et al., 2006; Tara et al., 2018). The ISI CV is the standard deviation of the ISI divided by the average ISI. The larger the ISI CV, the more irregular the firing and less precise the activity.

**Fig. 3.**
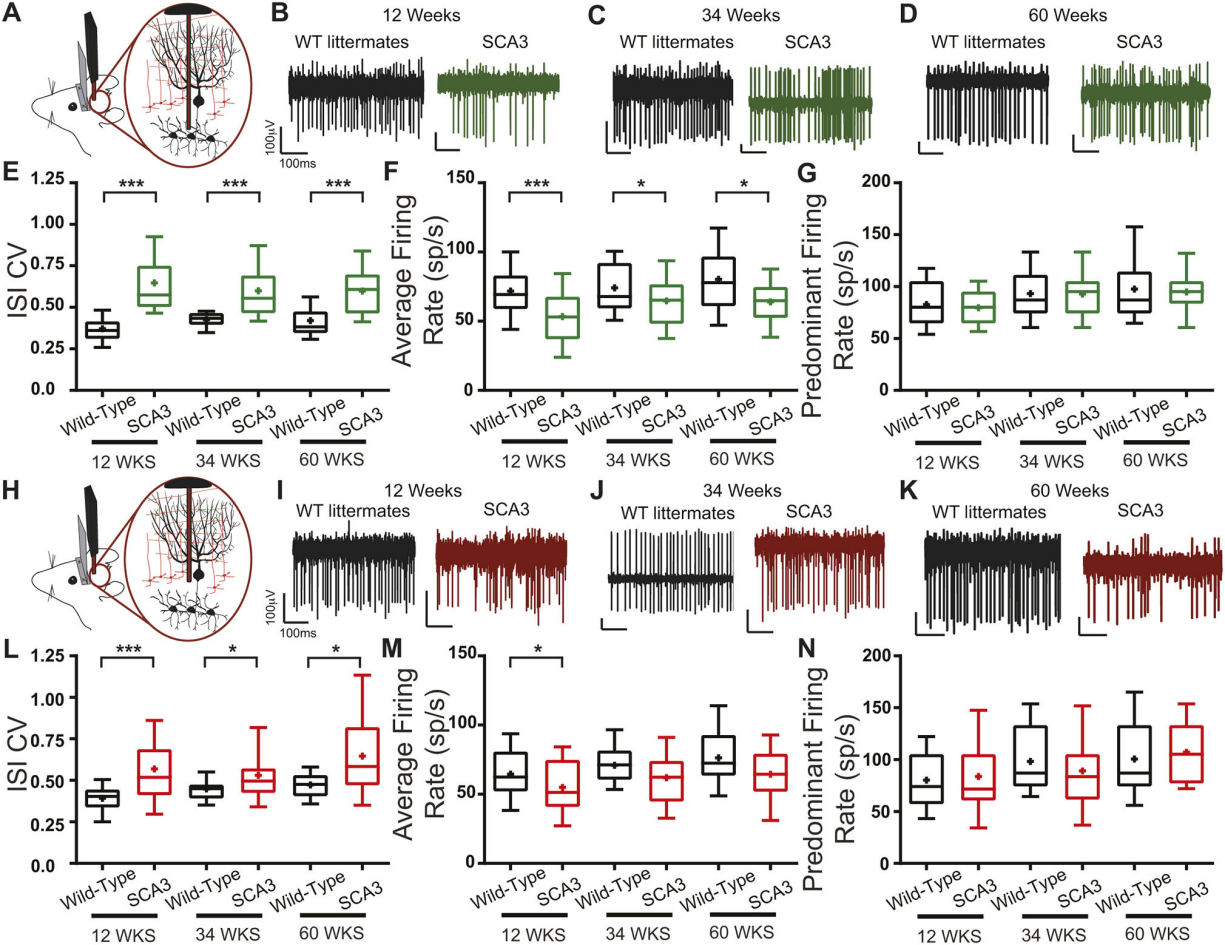
**Cerebellar neuronal dysfunction in SCA3 mice across disease progression.** (A,H) *In vivo* awake, head-fixed cerebellar DCN (A) and Purkinje cell (H) single-unit recordings were performed from SCA3 and wild-type mice at 12, 34 and 60 weeks of age. (B-D) Example DCN firing traces from wild-type (WT; black) and SCA3 (green) mice at 12 (B), 34 (C) and 60 (D) weeks of age. (E-G) DCN inter-spike interval coefficient of variation (ISI CV) (E) is increased in SCA3 mice, while the average firing rate (F) is decreased, with no change in predominant firing rate (G) for all ages tested. (I-K) Example Purkinje cell firing traces from wild-type (black) and SCA3 (red) mice at 12 (I), 34 (J) and 60 (K) weeks of age. (L-N) Purkinje cell ISI CV (L) is increased in SCA3 mice, while the average firing rate (M) is decreased or trends to decrease, with no change in predominant firing rate (N). DCN: 12 weeks: wild-type *N*=6, *n*=39, SCA3 *N*=8, *n*=33. 34 weeks: wild-type *N*=12, *n*=33, SCA3 *N*=16, *n*=65. 60 weeks: wild-type *N*=5, *n*=24, SCA3 *N*=3, *n*=25. Purkinje cells: 12 weeks: wild-type *N*=6, *n*=36, SCA3 *N*=8, *n*=32. 34 weeks: wild-type *N*=12, *n*=29, SCA3 *N*=16, *n*=40. 60 weeks: wild-type *N*=5, *n*=25, SCA3 *N*=5, *n*=25. *P<0.05, ***P<0.001. Unpaired, two-tailed Student's *t*-test (F, all; G, 12 weeks; M, all; N, 12 weeks, 60 weeks), Mann–Whitney test (E, all; G, 34 weeks, 60 weeks; L, all; N, 34 weeks).

In SCA3 mice, the ISI CV of DCN was increased, but not progressive, at 12, 34 and 60 weeks of age (12 weeks: wild-type 0.371±0.114, SCA3 0.648±0.199, *P*<0.0001; 34 weeks: wild-type 0.427±0.054, SCA3 0.599±0.170, *P*<0.0001; 60 weeks: wild-type 0.421±0.121, SCA3 0.599±0.152, *P*<0.0001) (Fig. 3E). DCN neurons had a decreased average firing rate (the reciprocal of the average ISI) at 12, 34 and 60 weeks of age in SCA3 mice (12 weeks: wild-type 71.9±18.9, SCA3 53.4±21.5, *P*=0.0002; 34 weeks: wild-type 74.1±19.8, SCA3 64.7±19.8, *P*=0.0359; 60 weeks: wild-type 80.3±26.0, SCA3 63.9±19.2, *P*=0.0154) (Fig. 3F). The decrease in average firing rate would be expected to result in a decreased ISI CV if the firing pattern had not changed. Therefore, with a decrease in firing rate, the increased irregularity, as reported by ISI CV, in SCA3 mice was greater than observed. Interestingly, although the ISI CV was increased at all time points, there was no progressive increase in the ISI CV, suggesting extracerebellar contributions to the progressive phenotype of SCA3 mice (Fig. S1A).

### Purkinje cell activity is irregular in SCA3 mice

Abnormal DCN firing can be caused by irregular firing of DCN neurons themselves, and from irregular input from neurons upstream of the output nuclei. Purkinje cells form the sole output of the cerebellar cortex and converge onto the DCN (Sillitoe et al., 2012). To determine whether, in SCA3 mice, Purkinje cell firing is altered, Purkinje cell activity was recorded *in vivo* in awake, head-fixed, stationary mice. Although it would be surprising to have progressive irregularity in the Purkinje cell activity that was not reflected in the DCN activity, the Purkinje cell activity was assessed for progressive neuronal dysfunction at all stages of disease.

At 12, 34 and 60 weeks, Purkinje cells in SCA3 mice had an increased ISI CV that was not progressive with age (12 weeks: wild-type 0.392±0.113, SCA3 0.570±0.261, *P*<0.0001; 34 weeks: wild-type 0.448±0.077, SCA3 0.531±0.183, *P*=0.0330; 60 weeks: wild-type 0.472±0.080, SCA3 0.646±0.262, *P*=0.0112), indicating irregularity of firing in SCA3 Purkinje cells compared with that of the wild-type mice (Fig. 3L). Similar to the DCN, Purkinje cells in 12-week-old SCA3 mice had a decreased average firing rate compared to wild types, with a trend to decrease at 34 and 60 weeks (12 weeks: wild-type 64.6±19.2, SCA3 54.9±20.5, *P*=0.0468; 34 weeks: wild-type 71.0±16.3, SCA3 61.9±20.7, *P*=0.0541; 60 weeks: wild-type 76.3±24.8, SCA3 64.4±21.2, *P*=0.0741) (Fig. 3M). With the observed decrease in average firing rate, this increased ISI CV likely represents a more pronounced irregularity of Purkinje cells. Taken together, these *in vivo* data demonstrated that Purkinje cell dysfunction was present at an early age, but not progressive.

### Synaptic and intrinsic components contribute to SCA3 Purkinje cell dysfunction

Both the spontaneous intrinsic activity of Purkinje cells and synaptic activity can contribute to the Purkinje cell irregular firing observed *in vivo*. Although motor-encoding activity was reduced by recordings in standing, stationary animals, synaptic input was still present *in vivo*. In addition, *in vitro* synaptic input can affect Purkinje cell firing through the spontaneous activity of neurons. Sensorimotor integration in the cerebellum occurs via the glutamatergic and GABAergic inputs onto Purkinje cells (Sillitoe et al., 2012). Recording Purkinje cell firing *in vitro* from acute cerebellar slices with synaptic transmission intact or blocked with inhibitors of fast GABAergic and glutamatergic transmission began to disentangle whether the Purkinje cell irregularity observed *in vivo* was from synaptic or intrinsic sources.

**Fig. 4.**
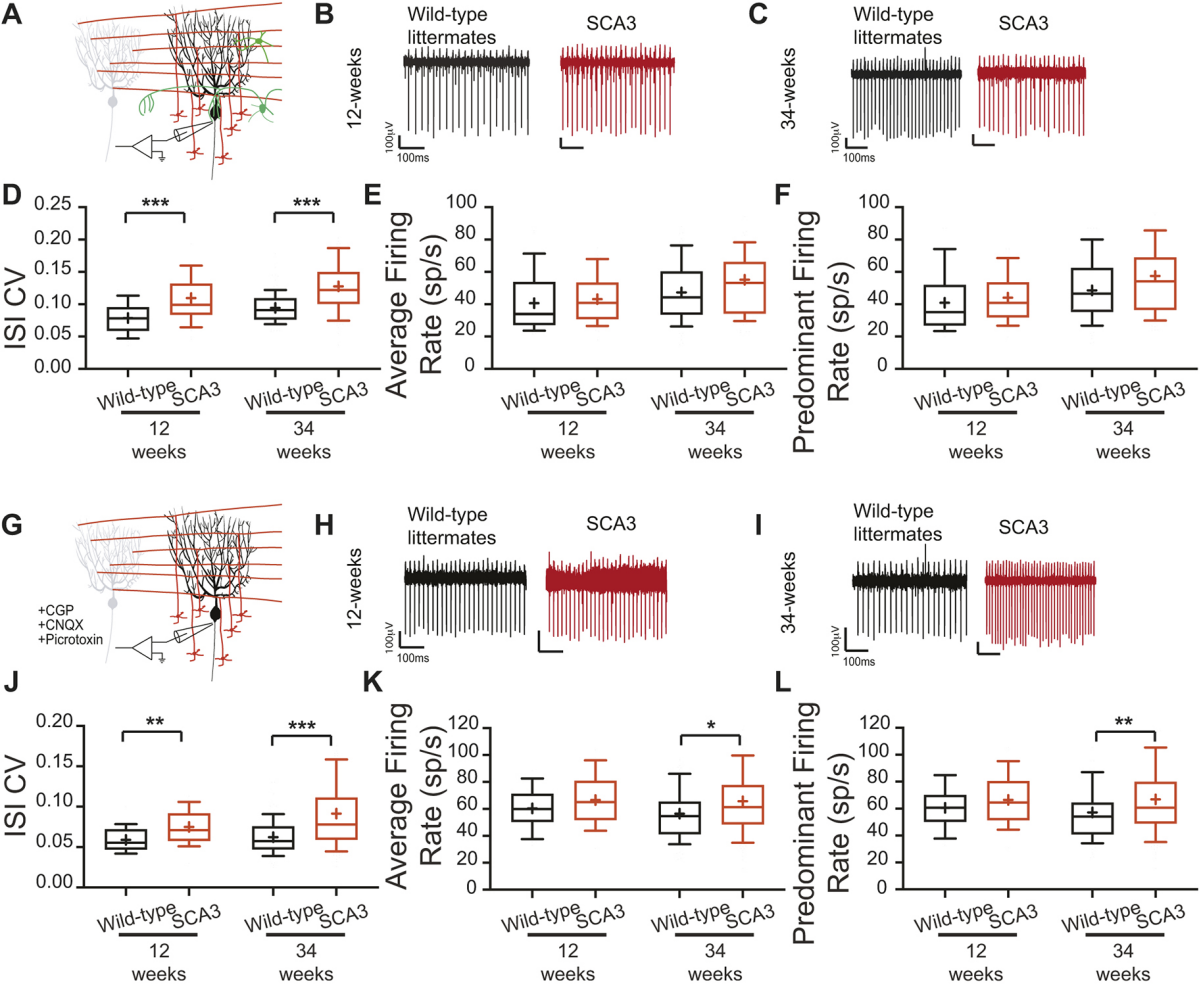
**Synaptic and intrinsic components to Purkinje cell irregularity.** (A-L) *In vitro* recordings were performed at 12 and 34 weeks of age, with synaptic transmission intact (A-F) and synaptic transmission blocked (G-L). Example Purkinje cell firing traces from wild-type (black) and SCA3 (red) mice at 12 weeks (B,H) and 34 weeks (C,I) of age. When synaptic transmission is intact (A-F), Purkinje cell ISI CV (C) is increased in SCA3 mice at both time points, with no change in average (E) and predominant (F) firing rate. When synaptic transmission is blocked (G-L), Purkinje cell ISI CV (J) is increased in SCA3 mice at both time points, with no change in average (K) and predominant (L) firing rate at 12 weeks, but an increase in both at 34 weeks. Synaptic transmission intact: 12 weeks: wild-type *N*=4, *n*=38; SCA3 *N*=4, *n*=50. 34 weeks: wild-type *N*=16, *n*=89; SCA3 *N*=17, *n*=91. Synaptic transmission blocked: 12 weeks: wild-type *N*=3, *n*=22; SCA3 *N*=3, *n*=29. 34 weeks: wild-type *N*=13, *n*=92; SCA3 *N*=15, *n*=121. *P<0.05, **P<0.01, ***P<0.001. Unpaired, two-tailed Student's *t*-test (K, 12 weeks; L, 12 weeks), Mann–Whitney test (D, all; E, all; J, all; K, 34 weeks; L, 34 weeks).

Acute sagittal cerebellar sections were obtained from 12- and 34-week-old mice, and extracellular single-unit recordings were made from visually identified Purkinje cells. Recordings were not made from 60 weeks of age due to technical difficulties maintaining mice to that age. When synaptic transmission was intact, the ISI CV of SCA3 Purkinje cells was significantly increased at both 12 and 34 weeks (12 weeks: wild-type 0.0787±0.0231, SCA3 0.1095±0.0449, *P*=0.0002; 34 weeks: wild-type 0.0942±0.0239, SCA3 0.1277±0.0439, *P*<0.0001) (Fig. 4D), whereas the average firing rate did not change (12 weeks: wild-type 40.7±17.7, SCA3 43.1±14.8, *P*=0.1919; 34 weeks: wild-type 47.3±17.6, SCA3 55.2±26.8, *P*=0.0743) (Fig. 4E). The increased ISI CV was not progressive, as expected by the *in vivo* data.

To determine whether intrinsic Purkinje cell dysfunction contributed to the increased ISI CV *in vitro*, fast synaptic transmission was blocked with GABA­_A_, GABA_B_ and AMPA (also known as GRIA)/kainate inhibitors. With synaptic transmission blocked, the ISI CV of Purkinje cells from SCA3 mice remained increased, but was not progressive, at 12 and 34 weeks (12 weeks: wild-type 0.0589±0.016, SCA3 0.0750±0.021, *P*=0.0040; 34 weeks: wild-type 0.0623±0.022, SCA3 0.0914±0.482, *P*<0.0001) (Fig. 4). The average firing rate of intrinsic Purkinje cell activity had a trend to increase at 12 weeks and was significantly increased at 34 weeks (12 weeks: wild-type 60.3±14.6, SCA3 66.5±19.0, *P*=0.2057; 34 weeks: wild-type 56.5±20.6, SCA3 65.6±28.0, *P*=0.0113) (Fig. 4D). The increased average firing rate suggested that the firing rate contributed to the increased ISI CV. Therefore, a difference in ISI CV when synaptic transmission was intact suggested that although both synaptic and intrinsic dysfunction occurred, the synaptic component was greater.

### Location dependence of SCA3 Purkinje cell irregularity

**Fig. 5.**
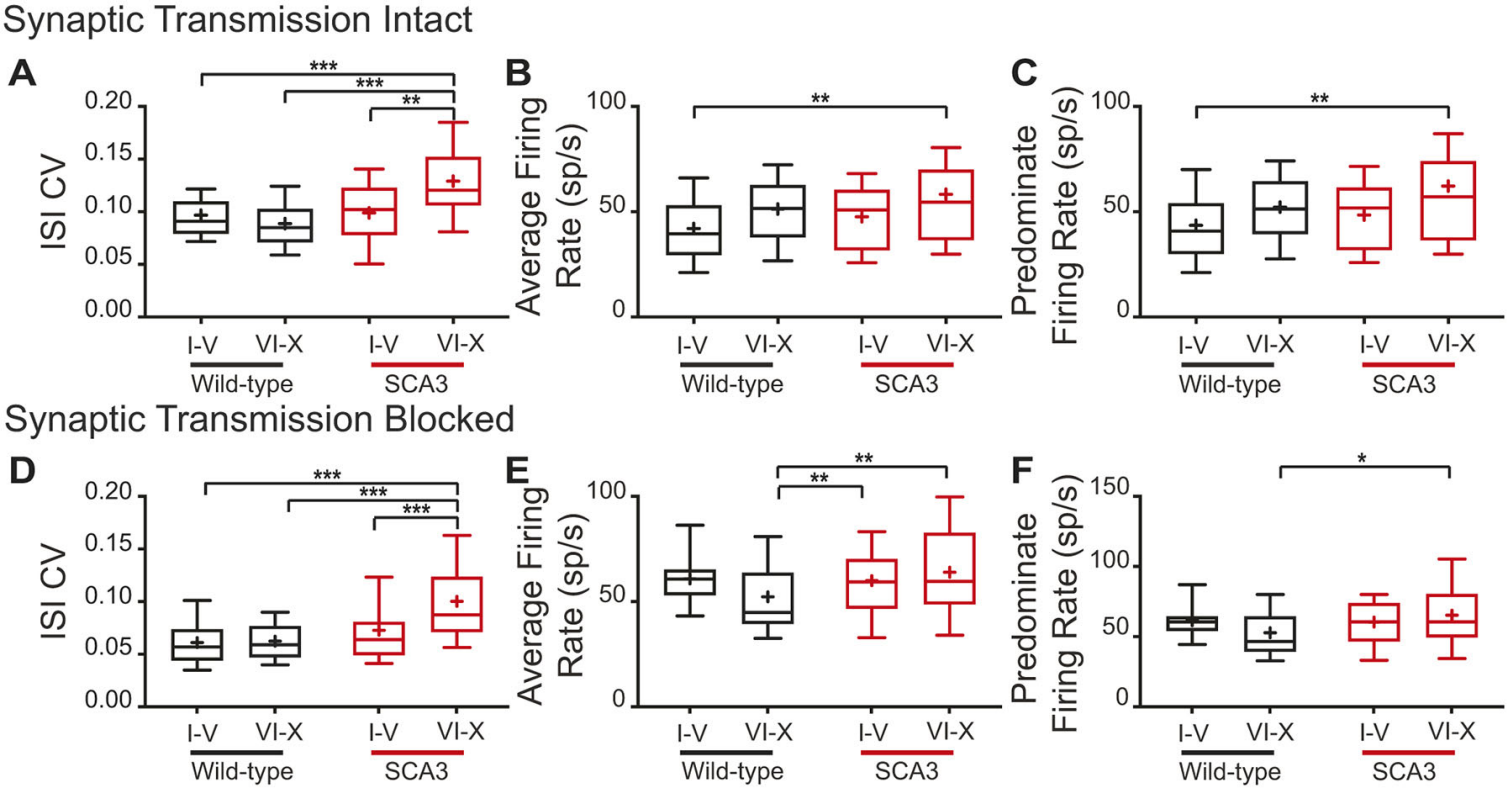
**SCA3 Purkinje cell dysfunction is heterogeneous.** (A-F) The *in vitro* Purkinje cell recordings from 34-week-old animals demonstrated dependence on location of recording. When synaptic transmission was intact (A-C), Purkinje cell ISI CV (A) was increased, with a small increase in average (B) and predominant (C) firing rates only in SCA3 lobules VI-X. This held true when synaptic transmission was blocked (D-F). The increased ISI CV (D), increased average firing rate (E) and increased predominant firing rate is specific to SCA3 lobules VI-X. Synaptic transmission intact: wild-type I-V: *N*=10, *n*=43; wild-type VI-X: *N*=10, *n*=33. SCA3 I-V: *N*=5, *n*=14; SCA3 VI-X: *N*=13, *n*=54. Synaptic transmission blocked: wild-type I-V: *N*=7, *n*=29; wild-type VI-X: *N*=12, *n*=59. SCA3 I-V: *N*=11, *n*=43; SCA3 VI-X: *N*=14, *n*=59. *P<0.05, **P<0.01, ***P<0.001. One-way ANOVA with Bonferroni correction for multiple comparisons.

Purkinje cells are not a homogenous population, with differential gene expression in stripes throughout the cerebellum (Cerminara et al., 2015). One differentially expressed gene, zebrin II (also known as *Aldoc*), is typically used as a guide for this heterogeneity, with lobules I-V being predominantly zebrin II negative, and lobules VI-X being predominantly zebrin II positive (Cerminara et al., 2015). With this rough generalization for gene expression, the Purkinje cell data from 34-week-old animals was sorted by the lobule in which it was located. In this preliminary analysis, SCA3 Purkinje cells in lobules I-V had no change in ISI CV (synaptic transmission intact: wild-type 0.097±0.023, SCA3 0.099±0.029, *P*>0.9999; synaptic transmission blocked: wild-type 0.061±0.024, SCA3 0.073±0.041, *P*>0.9999) (Fig. 5A,D). Nor was there a change in average firing rate in lobules I-V in SCA3 mice (synaptic transmission intact: wild-type 42.1±17.4, SCA3 47.7±15.8, *P*>0.9999; synaptic transmission blocked: wild-type 60.8±14.3, SCA3 60.0±17.5, *P*>0.9999) (Fig. 5B,E). In contrast, we found that SCA3 Purkinje cells in lobules VI-X had an increased ISI CV when synaptic transmission was intact (synaptic transmission intact: wild-type 0.089±0.025, SCA3 0.129±0.037, *P*<0.0001) (Fig. 5A) and when synaptic transmission was blocked (synaptic transmission blocked: wild-type 0.062±0.020, SCA3 0.100±0.044, *P*<0.0001) (Fig. 5D). In lobules VI-X, there was an increased average firing rate only when synaptic transmission was blocked (synaptic transmission intact: wild-type 51.3±15.9, SCA3 58.3±30.4, *P*>0.9999; synaptic transmission blocked: wild-type 52.3±21.8, SCA3 64.0±24.2, *P*=0.0076) (Fig. 5B,E). Thus, the aberrant synaptic and irregular intrinsic Purkinje cell activity in SCA3 mice is posterior lobule dependent.

## DISCUSSION

Here, we show that, early in disease, the activity of neurons in the DCN of SCA3 mice was irregular, with irregular Purkinje cell activity likely to be a contributing factor. The irregularity in the output of the cerebellum, the DCN, was noted in mice as early as 12 weeks postnatal; however, this irregularity was not progressive with age. Cerebellar Purkinje cells were also irregular *in vivo* as early as 12 weeks, but the irregularity was again not progressive with age. In *in vitro* experiments, we found that, in SCA3, both aberrant intrinsic firing and synaptic activity likely contribute to Purkinje cells dysfunction. The irregular firing in the DCN and Purkinje cells results in a decreased ability to accurately encode motor-related information. Therefore, despite the irregular firing not progressing with motor impairment, the early dysfunction, before any detectable DCN ataxin-3 intranuclear inclusion or increased neuronal death, suggested early and consistent cerebellar neuronal dysfunction likely driving the early motor phenotypes in SCA3. In patients, this early motor impairment can impact many aspects of their quality of life, as simple things such as dressing and eating require motor coordination. Therefore, with the goal of improving the quality of life of patients, this early Purkinje cell dysfunction could be a good therapeutic target for SCA3 patients.

### Early cerebellar dysfunction contributes to the SCA3 phenotype

Although the motor phenotype in SCA3 mice is progressive, the neuronal dysfunction in DCN and Purkinje cells appeared to be consistent from 12 to 60 weeks of age (Fig. S1). If increased cerebellar dysfunction was the primary factor in causing the progressive ataxia in SCA3, one might expect that, as the motor symptoms progressed, the neuronal dysfunction, or ISI CV, would also increase and correspond to the increased symptom severity (Tara et al., 2018). However, in SCA3, unlike pure cerebellar ataxias, the brainstem, spinal cord, muscles and other brain regions, such as the substantia nigra, are all affected (Woods and Schaumburg, 1972; Rosenberg et al., 1976; Romanul et al., 1977; Stefanescu et al., 2015; Hernandez-Castillo et al., 2018; Koeppen, 2018; Wang et al., 2020). Indeed, in this SCA3 mouse model, it is a combination of these factors that contribute to the presentation of motor symptoms. SCA3 mice have motor incoordination as early as 12 weeks of age and strength-related behavioral phenotypes that were previously shown as early as 6 weeks. By 60 weeks of age, mice are unable to properly support their body weight, often pulling their body to move instead of using proper ambulation (Teixeira-Castro et al., 2015). A potentially consistent cerebellar contribution to SCA3 ataxia has also been proposed based on studies using a different mouse model of SCA3 (Bushart et al., 2021). Additional studies will be needed to determine whether the dysfunctional cerebellum is driving other sites of dysfunction in the central nervous system, or whether these other areas undergo independent dysregulation. Given the ubiquitous expression of ataxin-3 in the nervous system of this mouse model (as in human patients), it might also be that the spinal cord/peripheral nervous system and striatum/substantia nigra-related mechanisms of dysfunction arise separately from the cerebellar mechanisms.

Despite the non-progressive nature of cerebellar irregular firing, the early initial dysfunction could be an appropriate therapeutic target. The cerebellum relies on the regular, spontaneous activity of the DCN to accurately encode movement-related information through rapid changes, up or down, in the firing rate of the DCN (Eccles, 1967; Thach, 1968). When the spontaneous activity of the cerebellum is no longer precise, the encoding of information is impaired, a feature apparent in other cerebellar ataxias (Walter et al., 2006; Alviña and Khodakhah, 2010b; Tara et al., 2018). The SCA3 mice have aberrant spontaneous and synaptic dysfunction of Purkinje cells, likely contributing to the altered DCN activity at an early stage of disease when motor incoordination is present. Targeting this neuronal activity therapeutically might provide a consistent, albeit partial, reduction in the severity of ataxia throughout the disease. In another SCA3 mouse model, when the mutant ataxin-3 was silenced with the use of antisense oligonucleotides, there was an improvement in the cerebellar deficits, and motor performance, of the SCA3 mice (Shakkottai et al., 2011; McLoughlin et al., 2018). This work reinforces the potential for cerebellar dysfunction to contribute to, and be a therapeutic target for, the motor dysfunction in SCA3. For patients, motor incoordination impacts most aspects of daily life. In fact, several components of the scale for assessment and rating of ataxia (SARA) depend on coordination, such as gait, finger-to-nose test, fast-alternating hand movements and finger chase (Schmitz-Hubsch et al., 2006; Jacobi et al., 2020). All of these measurements will affect daily tasks, such as dressing, grooming and eating. As SCA3 patients have a decreased quality of life early in disease (Bolzan et al., 2021), targeting this early dysfunction could alleviate some of these factors, potentially improving their quality of life.

### Purkinje cell dysfunction as one potential mechanism of SCA3 pathophysiology

In SCA3 patients, Purkinje cells show limited degeneration, if at all (Woods and Schaumburg, 1972; Rosenberg et al., 1976; Seidel et al., 2012a,b). Here, in a mouse model that does not show Purkinje cell ataxin-3 nuclear inclusions nor degeneration of Purkinje cells even at late disease stages (Rodríguez-Cueto et al., 2016), we found that Purkinje cells have irregular firing starting from the first stages of disease. One interesting feature of this irregularity was the dependence on location of the Purkinje cells within the cerebellum (Fig. 5). Expression of zebrin II correlates with expression of several genes that have the potential to influence the physiological properties of Purkinje cells, including *EAAT4* (also known as *Slc1a6*), *GABABr2* (also known as *Gabbr2*) and *mGluR1b* (also known as *Grm1*), among others (Brochu et al., 1990; Dehnes et al., 1998; Mateos et al., 2000; Larouche et al., 2006; Sarna et al., 2006; Chung et al., 2008; Sillitoe et al., 2009; Cerminara et al., 2015). Using zebrin II-linked gene expression patterns, Purkinje cells can be divided based on the modular organization of the cerebellum. The cerebellar vermis is divided anatomically into ten lobules, numbered I-X from anterior to posterior. Lobules I-V have Purkinje cells that are predominantly zebrin II negative, whereas lobules VI-X have more zebrin II positive Purkinje cells (Arancillo et al., 2015; Cerminara et al., 2015). Several different patterns of Purkinje cell degeneration have been noted in many models of cerebellar ataxia (Cerminara et al., 2015; Toscano Márquez et al., 2021). However, it is unclear how Purkinje cells are differentially affected by the different mutations leading to cerebellar ataxia. Further experiments are needed to directly correlate the changes in the firing pattern of Purkinje cells with zebrin II expression, and to delineate the reasons for this difference.

There are a few theories underlying SCA3 pathophysiology, ranging from the toxicity of protein aggregates/intranuclear inclusions to extensive cell death. One of the most consistently affected regions in pathological analysis of SCA3 patients is the DCN (Woods and Schaumburg, 1972; Rosenberg et al., 1976; Seidel et al., 2012a,b). In the SCA3 mouse model used here, the DCN show intranuclear aggregates without neuronal loss by 28 weeks, DCN volume loss by 42 weeks and DCN neuronal loss by 56 weeks (Silva-Fernandes et al., 2014; Rodríguez-Cueto et al., 2016; Duarte-Silva et al., 2018; Esteves et al., 2019). Here, we showed that SCA3 mice did not display intranuclear ataxin-3 inclusions or cell death in the DCN at 22 weeks of age, thus excluding the presence of these disease hallmarks at earlier ages, namely at 12 weeks, when we performed our analyses. Despite there being no pathology at 12 weeks, the DCN neurons *in vivo* had irregular activity. Although the possibility that this irregular activity is partially driven by DCN neurons themselves remains to be examined, similar to some other cerebellar ataxias, Purkinje cells in SCA3 mice have irregular firing. It is interested to note that erratic firing of Purkinje cells can lead to DCN degeneration, as in other mouse models of movement disorders, DCN pathology can be seen at the late stages of disease even when the causal mutation is isolated to Purkinje cells, or largely drives Purkinje cell specific irregularity (Todorov et al., 2012; Fremont et al., 2015). Together, this suggests the Purkinje cell dysfunction might not only be a contributor to the DCN irregularity but might lead to the DCN pathology observed later in disease. Additional studies are required to understand the mechanisms that cause the aberrant synaptic and intrinsic dysfunction of Purkinje cells. Understanding the contributors to cerebellar dysfunction might inform potential mechanisms driving all neuronal dysfunction in SCA3, which would allow the development of a multifaceted approach for treating this complex disease.

## MATERIALS AND METHODS

### Animals

Experiments were performed on 12- to 60-week-old CMVMJD135 (SCA3) mice with 140±10 CAG repeats, bred and genotyped at the Life and Health Science Research Institute (ICVS), School of Medicine, University of Minho, Braga, Portugal – ICVS/3B's – PT Government Associate Laboratory. Mice were housed at weaning in groups of five to six animals in filter-topped polysulfone cages 267×207×140 mm (370 cm^2^ floor area) (Tecniplast, Buguggiate, Italy), with corncob bedding (Scobis Due, Mucedola SRL, Settimo Milanese, Italy) in a specific pathogen-free animal facility. All animals were maintained under standard laboratory conditions: an artificial 12 h light/dark cycle (lights on from 08:00 to 20:00), with a room temperature of 21±1°C and a relative humidity of 50-60%. Mice were given a standard diet (4RF25 during the gestation and postnatal periods, and 4RF21 after weaning) (Mucedola SRL, Settimo Milanese, Italy) and water *ad libitum*. Health monitoring was performed according to Federation of European Laboratory Animal Science Association (FELASA) guidelines, confirming the specified pathogens status of sentinel animals maintained in the same animal room. All procedures were conducted in accordance with European regulations (European Union Directive 86/609/EEC). Animal facilities and the people directly involved in vertebrate animal experiments (A.N.-C.) as well as coordinating the research (P.M.) were certified by the Portuguese regulatory entity [Direcção Geral de Alimentação e Veterinária (DGAV)]. All protocols performed were approved by the Animal Ethics Committee of the Life and Health Sciences Research Institute, University of Minho and by the DGAV (reference 020317).

Mice were shipped to Albert Einstein College of Medicine and allowed at least 2 weeks of recovery before any experiments were conducted. All experiments were conducted in accordance with the guidelines set by the Institute of Animal Safety and Institute of at Albert Einstein College of Medicine under the senior investigator's (K.K.) Institutional Animal Care and Use Committee approved protocol. Mice were housed on a 12:12 h reversed light/dark cycle. Data were analyzed to examine any effect of repeat length on the electrophysiology, with no effect detected. As there have been no reported sex differences in these mice (Silva-Fernandes et al., 2010, 2014; Teixeira-Castro et al., 2011, 2015; Duarte-Silva et al., 2014, 2018; Esteves et al., 2015, 2019), only female mice were used for Figs 1 and 3, with all other figures including both male and female mice, with no difference between sex detected. For the 12-week time point, mice were 12-15 weeks of age. For the 34-week time point, mice were 34-40 weeks of age. For the 60-week time point, mice were 50-60 weeks of age. Mice were used for behavior first, followed by either *in vivo* or *in vitro* experiments.

### Behavior

All experiments were performed during the mouse's dark cycle. Mice were acclimated to the behavior room for at least 1 h before the start of all experiments. For all behavior experiments, the experimenter was blinded to the genotype of the mice.

#### Parallel rod floor test

Mice were assessed on the parallel floor rod test for 10-min sessions, once a day for 2 days (Kamens et al., 2005; Kamens and Crabbe, 2007). The trials were averaged to obtain total distance traveled and number of foot slips per animal. Ataxia ratio is defined as the number of foot slips (errors) divided by distance traveled (cm).

#### Disability score

Mice were assessed using the previously published disability score as follows: 0, normal motor behavior; 1, slightly slowed or abnormal movements; 2, mild impairments, limited ambulation unless disturbed; 3, moderate impairment, limited ambulation even when disturbed, frequent abnormal postures; 4, severe impairment, almost no ambulation, sustained abnormal postures; 5, prolonged immobility in abnormal postures (Weisz et al., 2005; Tara et al., 2018). To assess the disability score, mice were individually placed in an open field for 30 min. The videos were blinded, and a 30-s segment was selected in which the mouse was ambulating. The cropped video was sent to four viewers trained on the disability scale and blind to the genotype of the animal. These four scores were averaged to produce a score for each trial per mouse. The final score for each mouse was reported as the average of at least two trials from separate days.

### Neuropathology analysis

SCA3 and wild-type littermate mice (aged 22 weeks) were deeply anesthetized and perfused with PBS followed by 4% paraformaldehyde (PFA) in PBS. Brains were harvested and post-fixed overnight in a fixative solution. The following day, brains were transferred to sucrose 30% solution and further sliced in a vibratome. Free-floating sections (40 µm thick) were stained with Cresyl Violet or processed for immunohistochemistry with mouse anti-ataxin-3 (1:500, Millipore, 1H9, #MAB5360). Immunohistochemistry was performed using an ACUITYAdvanced Biotin Free Polymer DAB Kit (BioLegend, #931001) following the manufacturer's instructions. Pyknotic and healthy cells were quantified in the DCN (100% coverage; *N*=5 animals/group; *n*=2-8 slices/animal) and normalized for the total area (μm^2^) using a BX51 stereological microscope (Olympus) and Visiopharma integrator system software (Visiopharm). Representative staining images were acquired using optical microscopy (Olympus Widefield Upright Microscope BX61). All the samples were codified immediately after euthanasia (using numeric codes) and the quantifications were performed blindly.

### Electrophysiology

#### In vivo

Mice were implanted with a titanium bracket fixed to the skull with optibond (Kerr Dental) and charisma (Kulzer). A recording chamber was formed on the skull above the cerebellum with dental cement. Craniotomies for recordings were made at the following locations (anterior/posterior, medial/lateral): −6.2, ±1.75; and −7, ±0.25. Until time of recording, craniotomies were covered with silicone adhesive (KWIK-SIL, WPI). Surgery was performed on day 1. Recordings were performed on days 2-6. Mice were habituated to the head-fixed apparatus for at least 1 h before the recordings began each day. Recordings were only performed if the mouse was not moving in the apparatus. The same mouse was recorded from for a maximum of 3 days. From awake, head-fixed, non-moving mice, extracellular single-unit activity was recorded by advancing a tungsten electrode (Thomas Recordings, 2-3 MΩ) until Purkinje cell layers or DCN were detected. Purkinje cells were identified by location, presence of complex spikes and characteristic firing rate. DCN were identified by location and characteristic firing rate. Signals were filtered (200 Hz-20 kHz) and amplified (2000×) using a custom-built amplifier, and then digitized (20 kHz) using a National Instruments card (PCI-MIO-16-XE) with custom-written software in Labview. Waveforms were sorted offline using amplitude, energy and principal component analysis (Plexon offline sorter).

#### In vitro

All experiments were conducted blinded to the genotype of the mouse. Mice were anesthetized with isoflurane and rapidly decapitated. The brain was rapidly removed and placed in warm (35±2°C) artificial cerebral spinal fluid (ACSF): NaCl 125 mM, KCl 2.5 mM, NaHCO_3_ 26 mM, NaH_2_PO_4_ 1.25 mM, MgCl_2_ 1 mM, CaCl_2_ 2 mM, glucose 11 mM, pH 7.4 when gassed with 5% CO_2_:95% O_2_. The cerebellum was dissected, and 300 µm thick sagittal sections were made using a Campden Instruments 7000sz vibratome. Slices were kept in oxygenated ACSF at 35±2°C for 1 h and then kept at room temperature until use (up to 4 h). At the time of recording, slices were placed in a recording chamber perfused with warm (35±2°C) ACSF at 1.5 ml/min. Single-unit extracellular recordings were obtained from visually identified Purkinje cells with an upright microscope (Zeiss) using a home-made differential amplifier and glass pipette back-filled with ACSF. Where indicated, to isolate Purkinje cell intrinsic activity, perfusion ACSF contained 10 μM picrotoxin, 1 μM CGP55845 and 10 μM cyanquixaline (CNQX) to block GABA_A_, GABA_B_ and AMPA/kainate receptors, respectively. Signals were digitized (10 kHz) using a National Instruments card (PCI-MIO-16-XE) with custom-written software in Labview. Waveforms were sorted offline using amplitude, energy and principal component analysis (Plexon offline sorter), and output was analyzed using custom-written Labview software. For all experiments, each cell was held for a minimum of 5 min.

### Statistics

Data were graphed using 10-90 box plots, with the mean indicated by the plus sign. Unpaired, two-tailed Student's *t*-tests were used for all pairwise comparisons. All data were assessed for normalcy with the Shapiro–Wilk test, and non-parametric tests (Mann–Whitney) were conducted for all datasets that were not normally distributed. All data are reported in the text as ±s.d. For Fig. S1, nonlinear regression was performed with least squares regression, no weighting and a confidence interval of 95%. ‘*N*’ refers to number of mice; ‘*n*’ refers to number of cells. *P*<0.05 was considered significant.

## Supplementary Material

Supplementary information
