# Supplementary material for: Cerebellar neuronal dysfunction accompanies early motor symptoms in spinocerebellar ataxia type 3
Source: Dis Model Mech. 2022 Aug 5;15(8):dmm049514. doi: 10.1242/dmm.049514 (PMC9367011; doi:10.1242/dmm.049514)
Supplement: Supplementary information [file dmm-15-049514-s1.pdf]

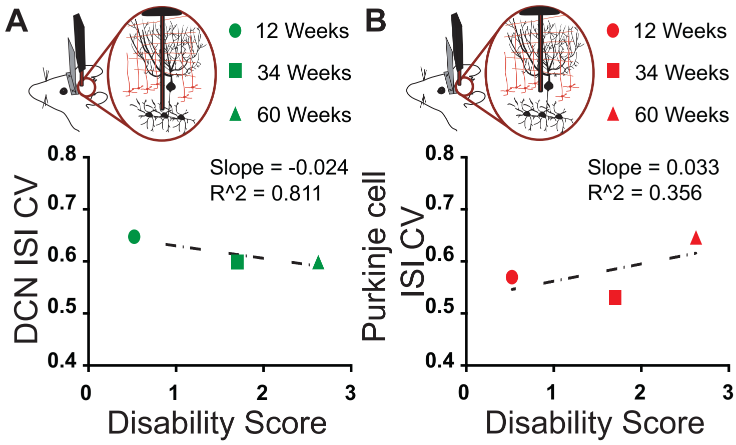

Fig. S1. The irregularity of SCA3 deep cerebellar neurons and Purkinje cells does not increase with the increased disability score. The *in vivo* recordings from SCA3 mice were compared to the disability score for 12, 34 and 60 weeks of age. Both the ISI CV from DCN (A) and from Purkinje cells (B) did not increase as the disability score increased. Nonlinear regression: least squares regression, no weighting, CI 95%. DCN: slope = -0.024, R^2 = 0.811. Purkinje cell: slope = 0.033, R^2 = 0.356.

Disease Models & Mechanisms • Supplementary information
